# SIRT6 Promotes Osteogenic Differentiation of Adipose-Derived Mesenchymal Stem Cells Through Antagonizing DNMT1

**DOI:** 10.3389/fcell.2021.648627

**Published:** 2021-06-22

**Authors:** Bo Jia, Jun Chen, Qin Wang, Xiang Sun, Jiusong Han, Fernando Guastaldi, Shijian Xiang, Qingsong Ye, Yan He

**Affiliations:** ^1^Department of Oral Surgery, Stomatological Hospital, Southern Medical University, Guangzhou, China; ^2^Department of Stomatology, Shunde Hospital, Southern Medical University, Foshan, China; ^3^Skeletal Biology Research Center, Department of Oral and Maxillofacial Surgery, Massachusetts General Hospital, Harvard School of Dental Medicine, Boston, MA, United States; ^4^The Seventh Affiliated Hospital, Sun Yat-sen University, Shenzhen, China; ^5^School of Stomatology and Medicine, Foshan University, Foshan, China; ^6^Center of Regenerative Medicine, Renmin Hospital of Wuhan University, Wuhan University, Wuhan, China; ^7^Laboratory of Regenerative Medicine, Tianyou Hospital, Wuhan University of Science and Technology, Wuhan, China

**Keywords:** sirtuin proteins 6, osteogenic differentiation, adipose-derived mesenchymal stem cells, NOTCH signaling, DNMT1

## Abstract

**Background:**

Adipose-derived stem cells (ADSCs) are increasingly used in regenerative medicine because of their potential to differentiate into multiple cell types, including osteogenic lineages. Sirtuin protein 6 (SIRT6) is a nicotinamide adenine dinucleotide (NAD)-dependent deacetylase that plays important roles in cell differentiation. NOTCH signaling has also been reported to involve in osteogenic differentiation. However, the function of SIRT6 in osteogenic differentiation of ADSCs and its relation to the NOTCH signaling pathways are yet to be explored.

**Methods:**

The *in vitro* study with human ADSCs (hADSCs) and *in vivo* experiments with nude mice have been performed. Alkaline phosphatase (ALP) assays and ALP staining were used to detect osteogenic activity. Alizarin Red staining was performed to detect calcium deposition induced by osteogenic differentiation of ADSCs. Western blot, RT-qPCR, luciferase reporter assay, and co-immunoprecipitation assay were applied to explore the relationship between of SIRT6, DNA methyltransferases (DNMTs) and NOTCHs.

**Results:**

SIRT6 promoted ALP activity, enhanced mineralization and upregulated expression of osteogenic-related genes of hADSCs *in vitro* and *in vivo*. Further mechanistic studies showed that SIRT6 deacetylated DNMT1, leading to its unstability at protein level. The decreased expression of DNMT1 prevented the abnormal DNA methylation of NOTCH1 and NOTCH2, resulting in the upregulation of their transcription. SIRT6 overexpression partially suppressed the abnormal DNA methylation of NOTCH1 and NOTCH2 by antagonizing DNMT1, leading to an increased capacity of ADSCs for their osteogenic differentiation.

**Conclusion:**

This study demonstrates that SIRT6 physical interacts with the DNMT1 protein, deacetylating and destabilizing DNMT1 protein, leading to the activation of NOTCH1 and NOTCH2, Which in turn promotes the osteogenic differentiation of ADSCs.

## Introduction

Adipose-derived stem cells (ADSCs), a type of mesenchymal stem cells, are being increasingly accepted as an attractive cell type for regenerative medicine because of their potential to differentiate into multiple cell types, including adipogenic, osteogenic, and chondrogenic lineages (Caplan, 2007; Bianco et al., 2008; Worthley et al., 2015). Owing to their osteogenic capacity and ease of acquisition and culture, human ADSCs (hADSCs) are considered a suitable cellular source for the regeneration of bone loss and fractures (Schubert et al., 2013; Heilmeier et al., 2016). However, the exact underlying mechanisms for osteogenic differentiation of ADSCs remain largely unknow.

Sirtuin protein 6 (SIRT6) is a nicotinamide adenine dinucleotide (NAD)+-dependent protein deacetylase involved in several important biological processes including genomic stability, transcriptional silencing, and DNA repair (Mostoslavsky et al., 2006; Kanfi et al., 2012). SIRT6 has a high affinity for chromatin and it universally deacetylates histone H3 lysine 9 (H3K9) and H3 lysine 56 (H3K56) in an NAD+-dependent manner (Kawahara et al., 2009). The deacetylation of telomeric H3K9 by SIRT6 is necessary for the function and structural stability of telomeric chromatin (Michishita et al., 2008).

Recent studies imply that SIR6 is associated with stem cell regulation. SIRT6 deacetylates the H3K56ac and H3K9ac of Oct4, Sox2, and Nanog at the promoter regions, and in turn controls embryonic stem cell differentiation (Etchegaray et al., 2015). SIRT6 deficiency leads the histone hyperacetylation at the imprinting control region of long non-coding RNA H19, and the activation of H19 delays neuronal differentiation (Zhang et al., 2018). These studies suggest the role of SIRT6 in the regulation of stem cell differentiation.

Previous studies reported SIRT1 could deacetylase DNA methyltransferases (DNMTs) and alter their activities (O’Hagan et al., 2008; Peng et al., 2011). DNA methylation is necessary for fundamental biological processes, including embryonic development, gene regulation, developmental potential of stem cells, and genomic imprinting (Jaenisch and Bird, 2003; Hayashi and Surani, 2009; Lee and Bartolomei, 2013), DNA methylation is catalyzed by DNMTs at 5 mC of CpG dinucleotides, DNMT1, DNMT3A, DNMT3B, and DNMT3L are responsible for *de novo* DNA methylation (Cedar and Bergman, 2009). Among them, DNMT1 often methylates newly replicated DNA and is regarded as a maintenance enzyme. DNMTs have been reported to involve in osteogenic differentiation of bone mesenchymal stem cell (Liu et al., 2016; Li et al., 2019). Furthermore, DNA methylation of the NOTCH family protein has been reported in stem cells (Chattapadhyaya et al., 2020; Wei et al., 2020). However, the association between SIRT6, DNMT1, and NOTCH signaling in ADSCs differentiation was yet to be elucidated.

In this study, we aim to investigate the effect of SIRT6 on the osteogenic differentiation of hADSCs and exam whether SIRT6 could modify the histone acetylation status of the DNMT1 and increasing the expression of NOTCH1 and NOTCH2. Our study may further strengthen the understanding of the mechanism underlying the osteogenic differentiation capacity of ADSCs.

## Materials and Methods

### Cell Lines and Reagents

The hADSCs were purchased from Cyagen Biosciences Inc. (Guangzhou, China). Briefly, the hADSCs were isolated and purified from fresh human adipose tissues donated from healthy adults less than 45 years of age after liposuction. The cells were cultured in Dulbecco’s modified Eagle’s medium (Gibco, Carlsbad, CA, United States) containing 10% fetal bovine serum and cultured at 37°C in a humidified atmosphere of 5% CO_2_. Osteogenic induction medium was purchased from SALILA (SALILA, Guangzhou, China). The medium was changed every 2 days. To inhibit DNA methylation, hADSCs were treated with 5 μmol/L 5-aza-2′-deoxycytidine (DAC, Sigma Aldrich, Munich, Germany). To inhibit NOTCH signaling, cells were treated with 2 μM DAPT (Selleck). Protein deacetylation was inhibited using 20 μM OSS_128167 (Selleck). For the cycloheximide (CHX)-chase assay, cells were treated with 100 g/mL CHX (Sigma-Aldrich, St. Louis, MO, United States) for the indicated hours in the absence or presence of 5 g/mL actinomycin D (Sigma-Aldrich, St. Louis, MO, United States) and western blot analysis was performed.

### Western Blot

The hADSCs were lysed on ice for 15 min using Radio Immunoprecipitation Assay (RIPA) lysis buffer (BeyoTime, Shanghai, China) supplemented with protease inhibitor cocktail (Roche, Basel, Switzerland). The protein concentration of the lysate was then measured using the BCA Protein Assay Kit (BeyoTime, Shanghai, China) according to the manufacturer’s protocol. Protein sample were subjected to SDS-PAGE and transferred to polyvinylidene difluoride membranes (Millipore, Billerica, United States), which were blocked with 5% fat-free milk and then incubated with specific primary antibodies overnight at 4°C An anti-rabbit-horseradish peroxidase (HRP) secondary antibody was added, and the staining was visualized using an enhanced chemiluminescence detection system (Millipore, Billerica, United States). The primary antibodies used in this study were as follows: SIRT6, RUNX2, SP7, COL1A1, NOTCH1, NOTCH2, NOTCH3, NOTCH4, JAG1, HEY1 (Abcam, Cambering, United Kingdom), HA, Flag, GAPDH (BeyoTime, Shanghai, China) DNMT1, Acetylated Lysine (Ac-K) (Cell Signaling Technology, Danvers, MA, United States).

### Quantitative Reverse Transcription (RT)-PCR

Total RNA was extracted using Trizol reagent (Invitrogen, Carlsbad, CA, United States). cDNA was synthesized using a Reverse Transcription Kit (Promega, Madison, WI, United States). cDNA was used for quantitative PCR reactions to determine the expression of specific genes with SYBR Green Real-Time PCR Master Mix Kit (Invitrogen, Carlsbad, CA, United States) according to the manufacturer’s instructions. GAPDH mRNA was applied as endogenous control. Primer sequences are shown in Supplementary Table 1. The total cycles for RT-PCR was set as 40.

### Lentivirus, siRNAs, and shRNAs Transfection

Lentivirus particles for the SIRT6 overexpression or knockdown of genes were purchased from GeneChem (GeneChem, Shanghai, China). The overexpressed plasmid was purchased from (Vigenebio, Jinan, China). The hADSCs in 24-well plates were infected with lentiviral vectors fixed with 10 mg/mL polybrene (GeneChem) in DMEM (Invitrogen, Carlsbad, CA, United States). Stable clones were selected using 0.5 μg/mL puromycin. Short hairpin RNAs (Supplementary Table 2) were synthesized by RiboBio (Guangzhou, China). hADSCs in 6-well plates were treated with 50 nM siRNAs or 4 μg shRNAs or 5 μg overexpression plasmid using Lipofectamine 3000 reagent (Invitrogen, Carlsbad, CA, United States) according to the manufacturer’s instructions and then harvested for assays.

### Alkaline Phosphatase Staining and Activity

Alkaline phosphatase (ALP) staining was performed in cells seeded in 24-well plates. After treatment with osteogenic differentiation medium for 7 days, the cells were fixed in 70% ethanol and incubated with a staining solution containing 0.1% naphthol AS-TR phosphate and 2% fast violet B (Sigma-Aldrich, St. Louis, MO, United States) for 1 h at room temperature. ALP activity was calculated quantitatively using a commercial ALP kit (Cell Biolab, San Diego, CA, United States) according to the manufacturer’s protocol.

### Alizarin Red Staining

Human ADSCs were incubated with osteogenic red S (pH 4.2, Sigma) for 10 min. For the quantitative assessment of the mineralization, the mineralized bone nodules were eluted with 10% (w/v) cetylpyridinium chloride (Sigma-Aldrich) for 1 h and analyzed by measuring the absorbance at 562 nm.

### Animal Experiments and Masson’s Trichrome Staining

Transplantation of *in vivo* experiment has been previously described (Jia et al., 2019). In brief, 4 weeks old nude mice were purchased from the Provincial Animal Center (Guangdong, China, No. 44002100023792). The mice were kept under a 12 h light/dark cycle in a drafty room at temperature of 22–26°C. Hydroxyapatite/tricalcium phosphate (HA-TCP; Sigma, St. Louis, MO, United States) was treated with 75% ethanol for sterilization for 12 h, washed by PBS for 6 times. Cut them into pieces and seeded with 200 ml of osteogenic differentiation-induced ADSCs suspension at a density of 5 × 10^6^ cells/ml. The mixed cells were subcutaneously inoculated into the right dorsal region of mice (5 mice per group). Four weeks later, the mice were sacrificed and the transplants were removed as previously described (Jia et al., 2019). The group implanted the mock vector was used as the control groups. The Animal experiments were approved by the Animal Care Committee of Southern Medical University. The samples were collected, fixed with 4% paraformaldehyde, and decalcified in 10% EDTA (pH 6.0) for 7 days. Paraffin sections were prepared and stained with hematoxylin and eosin, and Masson’s trichrome stain (Sigma-Aldrich, St. Louis, MO, United States) according to the manufacturers’ protocols.

### Luciferase Reporter Assay

The 2 kb region upstream of the transcription starting site of NOTCH1, NOTCH 2, and DNMT1 were synthesized and cloned upstream of the luciferase gene in the pmirGLO luciferase vector (GeneChem, Shanghai, China). hADSCs and 293T cells were treated with the indicated transfection particles using Lipofectamine 2000 (Invitrogen, Carlsbad, CA, United States). Promoter activity was measured using the luciferase assay kit (Promega, Madison, WI, United States) and normalized to the Firefly and Renilla luciferase activities 48 h after transfection.

### Co-immunoprecipitation

NP-40-containing lysis buffer supplemented with protease inhibitor cocktail was used to lyse cells (Roche, Basel, Switzerland), and the immunoprecipitated complexes were recovered with ChIP grade antibodies against acetylated lysine (Ac-K) (Cell Signaling Technology, Danvers, MA, United States), HA epitope (Beyotime, Shanghai, China), Flag epitope (Beyotime, Shanghai, China), rabbit immunoglobulin (Ig)G control antibodies (Sigma-Aldrich, St. Louis, MO, United States), which were incubated with protein A/G Sepharose beads (Santa Cruz, CA, United States), and then rinsed with wash buffer. The eluted immune complexes were denatured and subjected to western blot assay.

### Statistical Analysis

The data were presented as the mean ± SD. All data were analyzed using one- or two-way ANOVA with Bonferroni *post hoc* tests. SPSS 18.0 was used to perform all analyses. Statistical significance was defined as *p* < 0.05.

## Results

### SIRT6 Promoted Osteogenic Differentiation of hADSCs Both *in vitro* and *in vivo*

**FIGURE 1:**
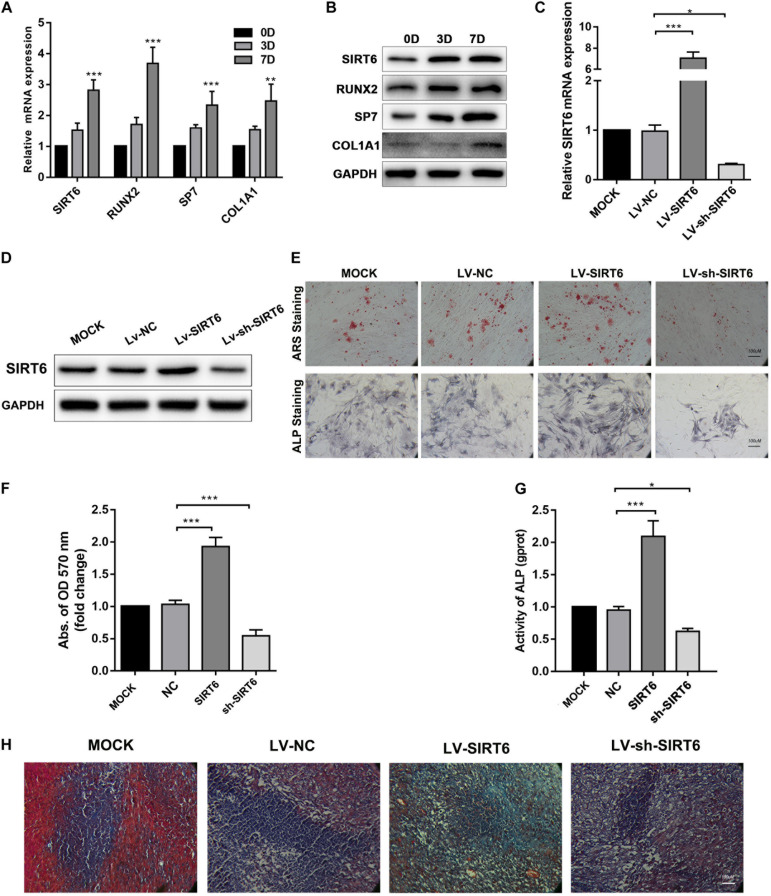
SITR6 promoted osteogenic differentiation of hADSCs. **(A,B)** Relative expression of SIRT6, RUNX2, SP7, and COL1A1 during osteogenic differentiation of hADSCs at day 0, 3, 7, determined by RT-qPCR and Western blot. **(C)** RT-qPCR analysis of SIRT6 in overexpression or knockdown hADSCs. **(D)** Western blot analysis of SIRT6 in overexpression or knockdown hADSCs. **(E)** Images of Alizarin red staining and ALP staining in SIRT6 overexpressed or silenced hADSCs. Cells were cultured in osteogenic differentiation media for 14 days. Alizarin red staining was quantified by spectrophotometry **(F)** Activity of ALP **(G)** (normalized to the NC groups). **(H)** SIRT6 promoted the osteogenesis of hADSCs *in vivo*. hADSCs loaded on HA-TCP were transplanted into the dorsal region of nude mice for 4 weeks. Then, sections of transplants were stained by Masson’s trichrome. Data were shown as means ± SD. *p < 0.05, **p < 0.01, ***p < 0.001.

In order to elucidate the role of SIRT6 in osteogenic differentiation, hADSCs were cultured under osteogenic inductive conditions. The hADSCs sustained their capacity for osteogenic differentiation, as indicated by gradually increased in the expression of osteogenic markers RUNX2, SP7 and ALP. The SIRT6 expression was increased after osteogenic induction (Figures 1A,B). Then, we constructed stable SIRT6-overexpression or knockdown hADSCs, confirmed by RT-qPCR and Western blot (Figures 1C,D). Alizarin red S (ARS) staining, ALP staining and activity assays revealed that upregulated SIRT6 promoted mineralized bone matrix formation and ALP activity in hADSCs, SIRT6 knockdown inhibited osteogenic differentiation in hADSCs (Figures 1E–G). To further evaluate the osteogenic function of SIRT6 in hADSCs. SIRT6 overexpressing hADSCs were cultured in osteogenic differentiation medium for 2 weeks and then implanted into immunocompromised mice subcutaneously using HA/TCP as a carrier. The hADSCs with SIRT6 overexpression exhibited significantly increased capacities to generate new bone as shown through Masson’s trichrome stain (Figure 1H). These results strongly suggest that SIRT6 promoted the osteogenesis of hADSCs both *in vitro* and *in vivo*.

### NOTCH Signaling Contributed to SIRT6-Mediated Promotion of Osteogenic Differentiation in hADSCs

**FIGURE 2:**
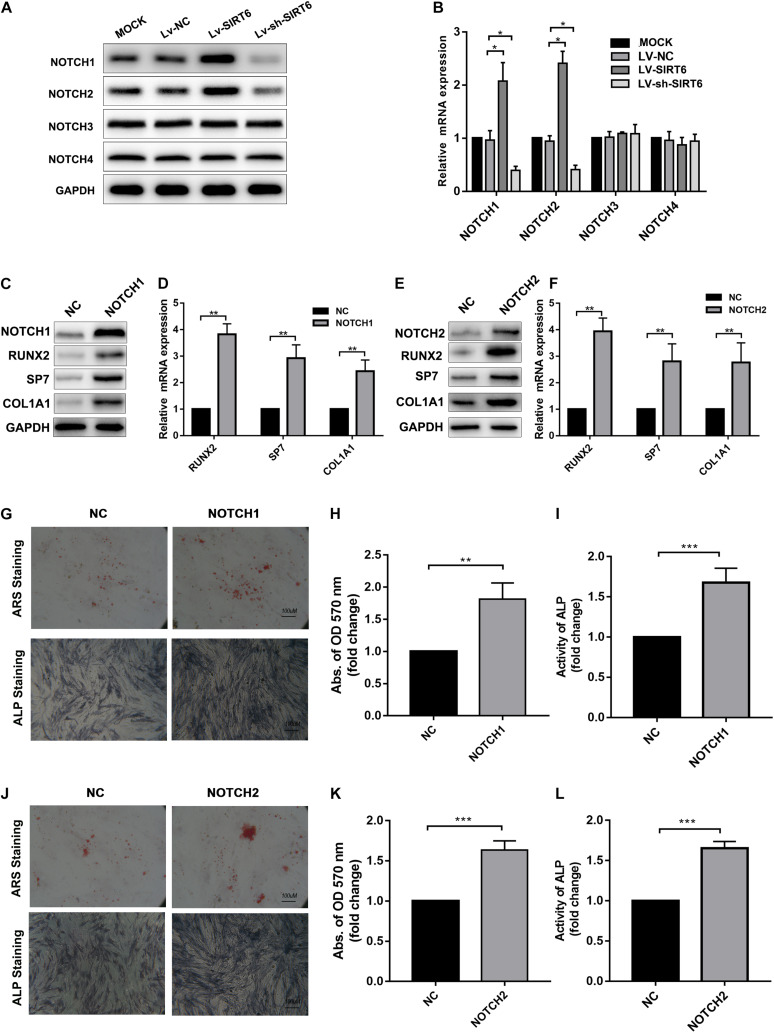
**(A,B)** Western blot and RT-qPCR showed the expression of NOTCH1, NOTCH2 was increased by SIRT6 overexpression and decreased by SIRT6 knockdown. **(C,D)** High expression of NOTCH1, RUNX2, SP7 and COL1A1 in NOTCH1 overexpressed hADSCs, was determined by Western blot and RT-qPCR. **(E,F)** Expression of NOTCH1, RUNX2, SP7, and COL1A1 in NOTCH2 overexpressed hADSCs, determined by Western blot and RT-qPCR. **(G–I)** Images of Alizarin red staining and ALP staining in NOTCH1 overexpressed hADSCs. Histograms show ALP activity and quantification of Alizarin red staining by spectrophotometry. **(J–L)** Images of Alizarin red staining and ALP staining in NOTCH2 overexpressed hADSCs. Histograms show ALP activity and quantification of Alizarin red staining by spectrophotometry. Data were shown as mean ± SD. *p < 0.05, **p < 0.01, ***p < 0.001.

NOTCH signaling has been reported to regulate bone mesenchymal cell differentiation (Dong et al., 2010).Also, previous studies revealed that the SIRT family may interact with NOTCH during differentiation (Bai et al., 2018; Okasha et al., 2020).Therefore, we hypothesized that NOTCH signaling was regulated by SIRT6 in the process of osteogenic differentiation in hADSCs. To test this hypothesis, we determined the expression of NOTCH family members in SIRT-overexpressed or silenced hADSCs, the results showed that the expression of NOTCH1 and NOTCH2 were increased following SIRT6 overexpression and decreased following SIRT6 knockdown both at mRNA and protein levels (Figures 2A,B). Then, we overexpressed NOTCH1 and NOTCH2 in hADSCs and found that the expression of osteogenic markers RUNX2, SP7, and ALP were increased consequently (Figures 2C–F). Mineralized bone matrix formation and ALP activity in hADSCs were also elevated in NOTCH1 and NOTCH2 overexpressing hADSCs as shown using ARS staining, ALP staining and activity assays (Figures 2G–L).

**FIGURE 3:**
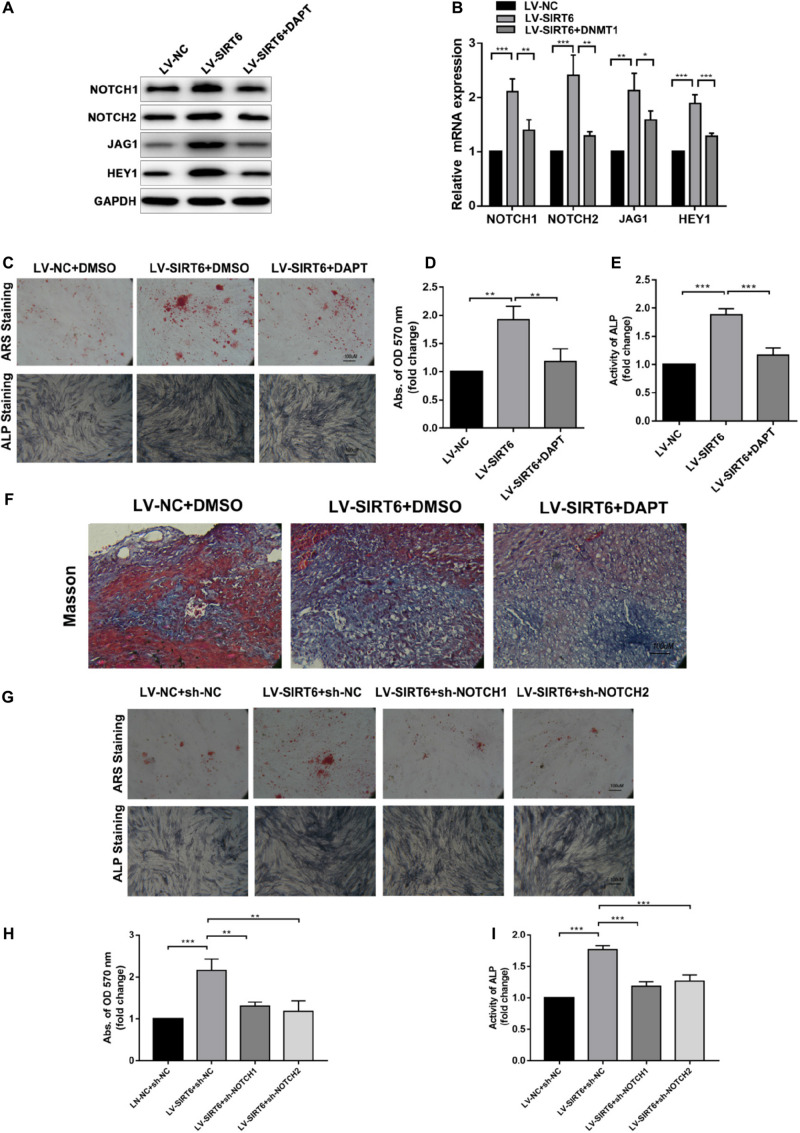
**(A,B)** SIRT6 overexpressed hADSCs were administered with DAPT, expression of NOTCH1, NOTCH2, JAG1, HEY1 were determined by Western blot and RT-qPCR. **(C–E)** SIRT6 overexpressed hADSCs were administered with DAPT and osteogenic differentiation medium for 14 days, Alizarin red staining and ALP staining showed mineralized nodule formation. ALP activity and quantification of Alizarin red staining by spectrophotometry were determined. **(F)** SIRT6 overexpressed hADSCs loaded on HA-TCP were transplanted into the dorsal region of nude mice, the mice were intraperitoneal injected with DAPT for 4 weeks. Then, the removed samples were measured by Masson’s trichrome staining. **(G–I)** SIRT6 overexpressed hADSCs were transfected with NOTCH1 or NOTCH2 shRNAs and cultured in osteogenic differentiation medium for 14 days, Alizarin red staining and ALP staining showed mineralized nodule formation. ALP activity and quantification of Alizarin red staining by spectrophotometry were determined. Data are shown as means ± SD. *p < 0.05, **p < 0.01, ***p < 0.001.

To identify the role of NOTCH signaling in SIRT6-mediated promotion of osteogenic differentiation, the NOTCH signaling inhibitor, DAPT, was applied in SIRT6-overexpressed hADSCs. DAPT treatment reversed the increase in the expression of NOTCH1, NOTCH2, JAG1, HEY1 induced by SIRT6 (Figures 3A,B). In addition, DAPT partially restrained the SIRT6-induced osteogenic differentiation capacity, as indicated by mineralized nodule formation and ALP activity (Figures 3C–E). In the *in vivo* experiment, DAPT also impaired the promoting effect of SIRT6 on hADSCs’ function in mineralized nodule formation, as illustrated using Masson’s trichrome staining (Figure 3F). To further confirm the role of NOTCH signaling in the regulation of hADSCs osteogenic differentiation, SIRT6-overexpression hADSCs were transfected with NOTCH1 or NOTCH2 shRNAs. ARS staining, ALP staining and activity assays demonstrated that silencing NOTCH1 or NOTCH2 could abolish the increased capacity of osteogenic differentiation by SIRT6 (Figures 3G–I). These data indicate that NOTCH signaling play an important role in the SIRT6-induced promotion of osteogenic differentiation in hADSCs.

### DNMT1 Suppressed Osteogenic Differentiation in hADSCs by Inducing Hypermethylation of the NOTCH1 and NOTCH2

**FIGURE 4:**
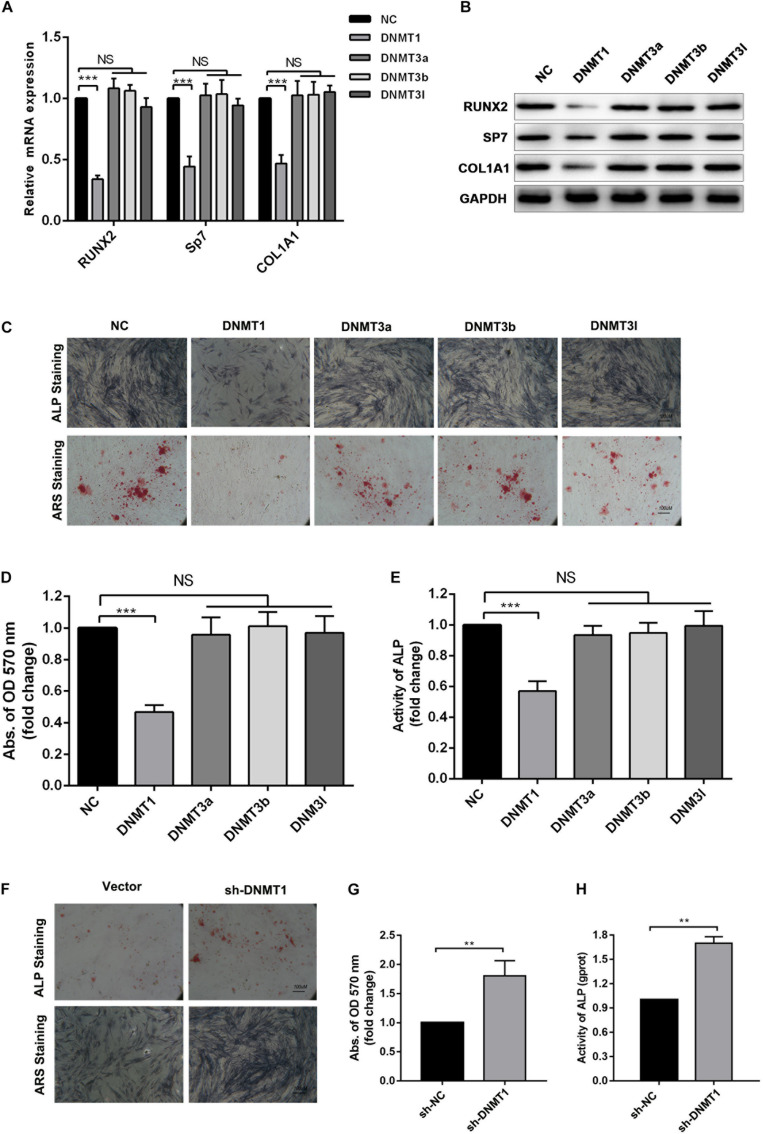
**(A,B)** Expression of RUNX2, SP7 and COL1A1 4 in DNMT1, DNMT3A, DNMT3B, DNMT3L overexpressed hADSCs were determined by Western blot and RT-qPCR. **(C–E)** hADSCs were transfected with DNMTs plasmid and cultured in osteogenic differentiation medium for 14 days, Alizarin red staining and ALP staining showed mineralized nodule formation, ALP activity and quantification of Alizarin red staining by spectrophotometry were determined. **(F–H)** DNMT1 silenced hADSCs were cultured in osteogenic differentiation medium for 14 days, Alizarin red staining and ALP staining showed mineralized nodule formation, ALP activity and quantification of Alizarin red staining by spectrophotometry were determined. Data are shown as means ± SD. **p < 0.01, ***p < 0.001.

Epigenetic regulation, such as DNA methylation, is essential for the self-renewal and differentiation of stem cells (Challen et al., 2011; Tsai et al., 2012). DNA methylation of CpG dinucleotides is regulated by DNMTs, including DNMT1, DNMT3A and DNMT3B and DNMT3L (Denis et al., 2011). We examined whether DNA methylation was involved in osteogenic differentiation of hADSCs. ShRNAs targeting DNMT1, DNMT3A and DNMT3B, and DNMT3L were transfected into hADSCs and the capacity of osteogenic differentiation were assessed. We found DNMT1, rather than DNMT3A and DNMT3B or DNMT3L, was verified as a potential regulator in osteogenic differentiation of hADSCs (Figures 4A,B). Following overexpression of DNMTs, only DNMT1 significantly decreased the expression of osteogenic marker and impaired the capacity of osteogenic differentiation in hADSCs, as confirmed through ARS staining, ALP staining, and activity assays (Figures 4C–E). DNMT1 silencing increased mineralized nodule formation and ALP activity *in vitro* (Figures 4F–H).

**FIGURE 5:**
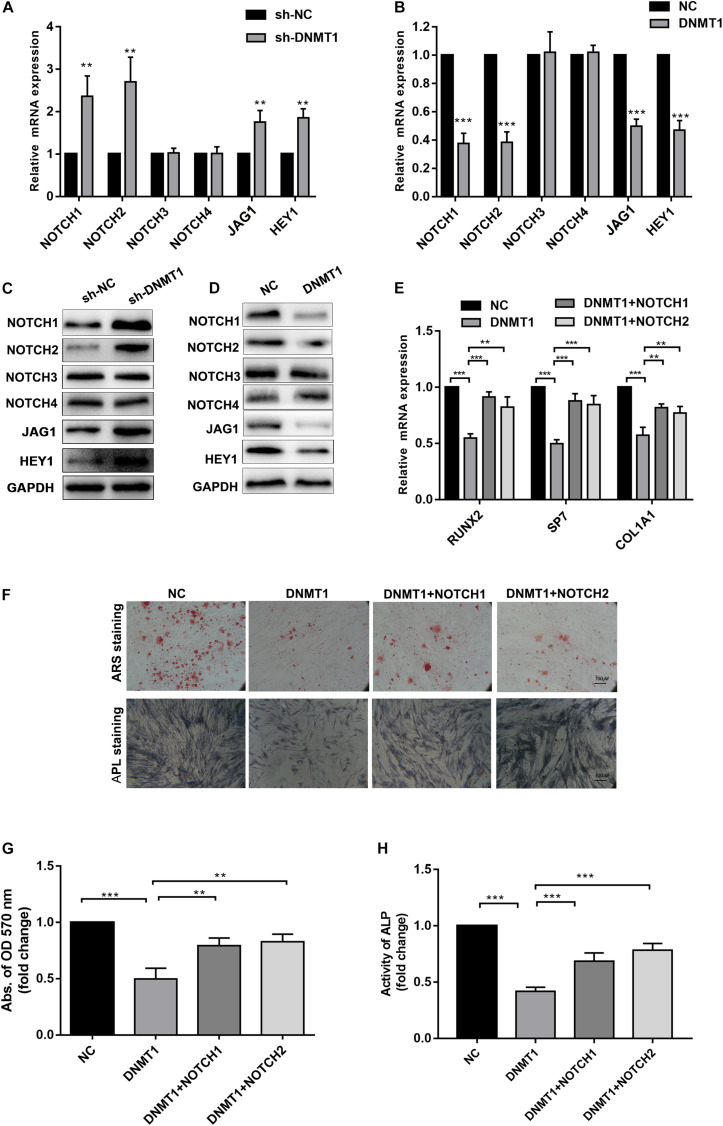
**(A,B)** Human ADSCs were transfected with DNMT1 shRNA plasmid, expression of NOTCH1, NOTCH2, NOTCH2, NOTCH3,NOTCH4, JAG1, HEY1, were determined by Western blot and RT-qPCR. **(C,D)** hADSCs were transfected with DNMT1 overexpression plasmid, expression of NOTCH1, NOTCH2, NOTCH2, NOTCH3, NOTCH4, JAG1, HEY1, were determined by Western blot and RT-qPCR. **(E)** DNMT1 overexpressed hADSCs were transfected with NOTCH1 or NOTCH2 overexpression plasmids, expression of RUNX2, SP7, and COL1A1 were determined by Western blot and RT-qPCR. **(F–H)** DNMT1 stably overexpressed hADSCs were transfected with NOTCH1 and NOTCH2. After differentiation medium for 14 days, Alizarin red staining and ALP staining showed mineralized nodule formation, ALP activity and quantification of Alizarin red staining by spectrophotometry were determined. Data are shown as means ± SD. **p < 0.01, ***p < 0.001.

To gain insights into weather the hypermethylation of the NOTCH1 promoters was regulated by DNMT1, the expression of NOTCH1, NOTCH2, the upstream ligand of NOTH signaling JAG1, and the import downstream target HEY1 were detected. The result implied that NOTCH1, NOTCH2, JAG1, and HEY1 expression was remarkably increased in DNMT1 silenced hADSCs at both transcription and protein levels (Figures 5A,B). In contrast, DNMT1 overexpression suppressed the NOTCH signal (Figures 5C,D). Moreover, when co-transfection with NOTCH1 or NOTCH2 overexpression plasmid in DNMT1-overexpressed hADSCs partially rescued the DNMT1 induced impairment of osteogenic differentiation hADSCs, as indicated by expression of RUNX2, and SP7 the mineralized nodule formation capacity and ALP activity (Figures 5E–H). These data showed that DNMT1 could promote osteogenic differentiation of hADSCs by inducing the hypermethylation of the NOTCH1 and NOTCH2.

### SIRT6 Suppressed the DNMT1 Transcription and Deacetylated the DNMT1 Protein via Physical Interactions

**FIGURE 6:**
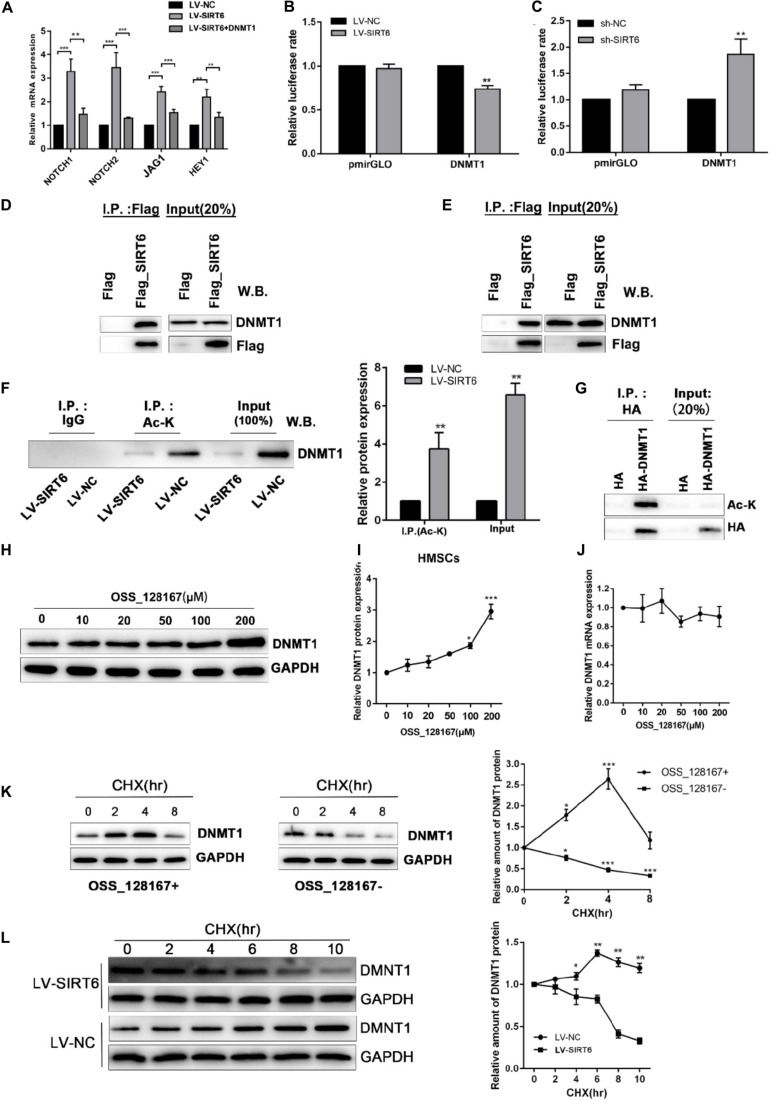
**(A)** SIRT6 overexpressed hADSCs were transfected with DNMT1 plasmid, the expression of NOTCH1, NOTCH2, NOTCH2, JAG1, HEY1 were determined by RT-qPCR. **(B,C)** Promoter activity was assessed for DNMT1 in SIRT6 overexpressed of silenced hADSCs (*n* = 3). **(D,E)** Immunoprecipitation (IP) analysis was performed in Flag-SIRT1-overexpressing hADSCs and 293T cells. A stable empty vector transfected cell line was established as control group. **(F)** IP analysis was performed with an acetylated lysine (Ac-K) antibody using flag-SIRT6-overexpressing hADSCs nuclear extracts. DNMT1 proteins levels in the Ac-K IP is shown as mean ± SEM. **(G)** HA-tagged Dnmt1 proteins were immunoprecipitated (IP) using an HA antibody. Acetylated proteins in the IP products were detected by western blot. **(H,I)** The change of DNMT1 protein expression are shown during treatment of OSS_128167. **(J)** The change of DNMT1 mRNA expression is shown during treatment of OSS_128167. (K) DNMT1 proteins stability in hADSCs in the present or absent of OSS_128167 was measured using a cycloheximide (CHX)-chase assay (100 mg/mL CHX) for the indicated hours. **(L)** Stability of the DNMT1 proteins in SIRT6 overexpressed or control hADSCs was measured using a CHX-chase assay involving 100 mg/mL CHX for the indicated hours. Data are shown as means ± SD. *p < 0.05, **p < 0.01, ***p < 0.001.

Based on the above results, we proposed a hypothesis that regulation of DNMT1 expression by the SIRT6 protein was responsible for the inhibition of NOTCH signaling in osteogenic differentiation of hADSCs. Consistent with the above result, DNMT1 overexpression could abolish the SIRT6-induced increase of NOTCH1, NOTCH2, JAG and HEY1 expression (Figure 6A). In order to assess whether SIRT6 could suppress DNMT1 transcription, a luciferase vector was constructed upstream of the transcription starting site of DNMT1 was constructed. We confirmed that SIRT6 overexpression suppressed the DNMT1 promoter activity, in contrast, SIRT6 knockdown increased the luciferase activity (Figures 6B,C). As reported, the SIRT1 protein physically interacted with DNMT3L and regulates its activity by protein deacetylation. In this study, we assessed the potential physical interactions between SIRT6 and DNMT1 proteins. Co-immunoprecipitation assay was performed in hADSCs and 293T cells with flag tagged SIRT6 protein. Endogenous DNMT1 protein was identified in the SIRT6 protein complex (Figures 6D,E).

To identify whether SIRT6 deacetylate DNMT1, we examined the acetylation status of the DNMT1 protein in hADSCs via immunoprecipitation assays. The results demonstrated that an amount of DNMT1 protein was acetylated in hADSCs, and acetylated DNMT1 was found at a remarkably lower level in SIRT6 overexpressed hADSCs (Figures 6F,G). These results suggest that DNMT1 protein could be acetylated by SIRT6 in hADSCs.

**FIGURE 7:**
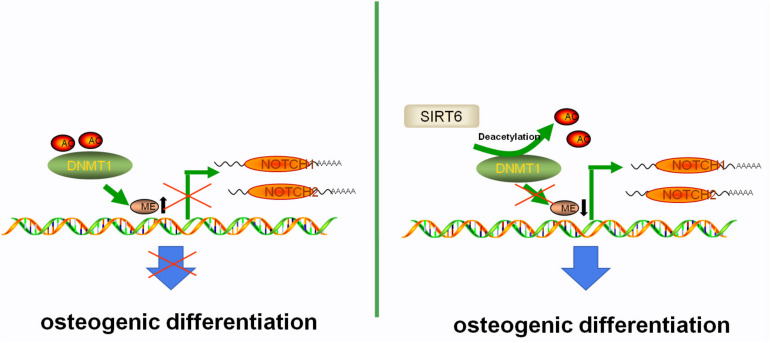
The proposed mechanism of SIRT6-induced osteogenic differentiation of adipose-derived mesenchymal stem cells. SIRT6 tightly regulates the level of NOTCHs by antagonizing the DNMT1 protein at the level of protein stability. Abnormal DNA methylation status of NOTCHs caused by DNMT1 in SIRT6 deficiency impairs the osteogenic differentiation potential of ADSCs. In the present of SIRT6, the histone acetylation status of the DNMT1 was upregulated, leading to the increased expression of NOTCH1 and NOTCH2, which further promotes the osteogenic differentiation capability of ADSCs.

Next, we determined the biological significance of the acetylation status in DNMT1, SIRT6 overexpressing hADSCs were administrated with OSS_128167, a chemical inhibitor of SIRT6. Treatment with OSS_128167 increased the protein and mRNA levels of DNMT1 (Figures 6H–J). Then, we performed a CHX chasing assay to check the stability of the DNMT1 protein with or without OSS_128167 treatment. As shown in Figure 6K, blocking the SIRT6 deacetylase activity dramatically increased the stability of DNMT1. In addition, the DNMT1 protein stability was weaker in SIT6 overexpressing hADSCs than in control cells (Figure 6L). These results indicate that SIRT6 regulates DNMT1 protein stability via a posttranslational modification mechanism. A diagram was show in Figure 7 to visualize the detail mechanism in our study.

## Discussion

SIRT6 has been identified as an NAD1-dependent deacetylase and it is crucial for genome stability, telomere integrity, and life span (Michishita et al., 2005). Recently SIRT6 deficiency was found to be involved in several bone metabolism disease, such as osteoporosis and osteoarthritis, which may due to the broken balance of bone homeostasis (Sugatani et al., 2015; Zhang et al., 2018). Further, knockdown of SIRT6 accelerated the aging of the marrow mesenchymal stem cells (BMSCs) while its overexpression could promote the osteogenic differentiation (Zhai et al., 2016). SIRT6 reported to promote the osteogenic differentiation via modifying the BMP signaling in a PCAF dependent manner (Zhang et al., 2017). In the present study, we demonstrated that SIRT6 expression was increased after osteogenic induction of hADSCs, SIRT6 overexpression significantly increased the expression of osteogenic markers and promoted mineralized bone matrix formation and ALP activity. The effects of SIRT6 overexpression on osteogenic differentiation could be offset by NOTCH1/2 shRNAs or DAPT. The expression of NOTCH1 and NOTCH2 was epigenetic regulated by DNMT1. DNMT1 impaired the capacity of osteogenic differentiation in hADSCs as confirmed by ARS staining, ALP staining and activity assays, which could be rescued by through SIRT6 overexpression as well as NOTCH1 and NOTCH2 plasmid transfection. SIRT6 promoted osteogenic differentiation by preventing abnormal DNA methylation of NOTCH1 and NOTCH2 via antagonizing DNMT1.

Altered DNA methylation of the NOTCH family protein has been observed in stem cells (Liu et al., 2015; Yan et al., 2018). Consistent with these reports, we identified that NOTCH1 and NOTCH2 were regulated by DNMT1. However, we were unable to explain why DNMT1 selectively hypermethylated NOTCH family members. DNA methylation of these two genes resulted in increased ALP activity, enhanced mineralization and elevated expression level of osteogenic-related genes. Inhibition of NOTCH signaling via silencing JAG1 caused an impairment of osteogenic differentiation (Qu et al., 2017). Tetrahedral DNA nanostructures could dramatically enhance the proliferation and osteogenic differentiation of dental pulp stem cells by upregulating the NOTCH signaling (Zhou et al., 2018). Similar mechanism was reported in BMSCs and it seems that microenvironment plays an important role (Chen et al., 2013; Xie et al., 2018). In this study, overexpression of NOTCH1 and NOTCH2 promoted the osteogenic differentiation of hADSCs and DNMT1 rather than DNMT3A, DNMT3B or DNMT3L could restrain this effect. Hence, we speculated that the specificity of SIRT6 targets could be determined by their association with the DNMT1 protein. In our study, the SIRT6 protein deacetylated and destabilized DNMT1 protein via physical interaction. Peng et al. Reported that DNMT3L is a substrate of the SIRT1 protein (Peng et al., 2011), and DNMT3L expression and the protein stability were regulated by SIRT1 (Heo et al., 2017). Here, we identified that SIRT6 protein strongly interacted with DNMT1. DNMT1 overexpression significantly suppressed NOTCH1 and NOTCH2 and impaired the osteogenic differentiation. This suggested that DNMT1, a crucial target of SIRT6, involved in regulation of DNA methylation of NOTCH1 and NOTCH2 in hADSCs. Thus, SIRT6 might affect distinct downstream targets via DNMT1, which regulated the DNA methylation status of this gene. Here, we revealed that DNMT1 is a substrate of SIRT6 deacetylase in hADSCs and identification of the proteins directly modified by SIRT6 would be helpful in understanding the biological significance of the SIRT6-DNMT1 complex in the multi-directional differentiations of hADSCs (Figure 7). Our data reemphasized the import role for SIRT6 in regulating osteogenesis found in other marrow mesenchymal stem cells, while also establish a previously unconfirmed underlying mechanism. The new mechanism we discovered may support a potential therapeutic application through a SIRT6 agonist or DNA methyltransferase inhibitor in the bone metabolism diseases.

## Conclusion

In our study, we identified the regulation of osteogenic potential of hADSCs composed of SIRT6/DNMT1/NOTCHs axis. SIRT6 increased the levels of NOTCH1 and NOTCH2 via acetylated DNMT1, which could hypermethylated NOTCH1 and NOTCH2. Our study demonstrated the promotion of SIRT6 in osteogenic differentiation and its potential role in the treatment of osteogenesis disorders.

## Data Availability Statement

The original contributions presented in the study are included in the article/Supplementary Materials, further inquiries can be directed to the corresponding authors.

## Ethics Statement

The animal study was reviewed and approved by Animal Ethics Committee of the Southern Medical University.

## Author Contributions

BJ, YH, and QY conceived the research idea and designed the whole experimental plan. JC, QW, and XS isolated and characterized the adipose-derived mesenchymal stem cells. BJ, JH, and SX performed the animal study. BJ and YH analyzed the *in vitro* and *in vivo* results and discussed the findings with YH. BJ wrote the initial draft of the manuscript. YH and QY revised the manuscript. FG provided constructive suggestion on the discussion and proofread the language of the manuscript. All authors have approved the final version of this manuscript.

## Conflict of Interest

The authors declare that the research was conducted in the absence of any commercial or financial relationships that could be construed as a potential conflict of interest.

## Abbreviations

ADSCs: Adipose-derived stem cells
ALP: Alkaline phosphatase
SIRT6: Sirtuin proteins 6
DNMTs: DNA methyltransferases
ARS: Alizarin red S
CHX: cycloheximide.
