# Supplementary material for: SIRT6 Promotes Osteogenic Differentiation of Adipose-Derived Mesenchymal Stem Cells Through Antagonizing DNMT1
Source: Front Cell Dev Biol. 2021 Jun 22;9:648627. doi: 10.3389/fcell.2021.648627 (PMC8258422; doi:10.3389/fcell.2021.648627)
Supplement: Supplementary file 1 [file Data_Sheet_1.docx]

SIRT6 promotes osteogenic differentiation of adipose-derived mesenchymal stem cells through activating notch signaling via antagonizing DNMT1

Bo Jia^1,2#^, Jun Chen^1#^, Qin Wang^1^, Xiang Sun^1^, Jiusong Han^1^, Fernando Guastaldi^3^, Shijian Xiang^4^, Qinsong Ye^#5,6*^, Yan He^7*^

*1 Department of Oral Surgery, Stomatological Hospital, Southern Medical University, Guangzhou, China,*

*2 Department of Stomatology, Shunde Hospital, Southern Medical University, Foshan, China,*

*3 Skeletal Biology Research Center, Department of Oral and Maxillofacial Surgery, Massachusetts General Hospital, Harvard School of Dental Medicine, Boston, MA, United States,*

*4 The Seventh Affiliated Hospital, Sun Yat-sen University, Shenzhen, China,*

*5 School of Stomatology and Medicine, Foshan University, Foshan, China,*

*6 Center of Regenerative Medicine, Renmin Hospital of Wuhan University, Wuhan University, Wuhan, China,*

*7 Laboratory of Regenerative Medicine, Tianyou Hospital, Wuhan University of Science* *and Technology, Wuhan, China*

Supplementary Material

# Supplementary Figures and Tables

**Supplementary Table 1. Sequences of PCR primers used in this study**

| SIRT6 | Forward(5’-3’) | CCCACGGAGTCTGGACCAT |
| --- | --- | --- |
|  | Reverse(5’-3’) | CTCTGCCAGTTTGTCCCTG |
| RUNX2 | Forward(5’-3’) | CTGGCCTTCCACTCTCAGTAA |
|  | Reverse(5’-3’) | ACTGGCGGGGTGTAAGTAAAG |
| SP7 | Forward(5’-3’) | GGCGTCCTCCCTGCTTGA |
|  | Reverse(5’-3’) | CCTGCTTTGCCCAGAGTTGT |
| COL1A1 | Forward(5’-3’) | TGCTCGTGGAAATGATGGTG |
|  | Reverse(5’-3’) | GGAGCACCATTGGCACCTTT |
| NOTCH1 | Forward(5’-3’) | GCCTGAATGGCGGGAAGT |
|  | Reverse(5’-3’) | GGTGGGCAGTGGCAGATGTA |
| NOTCH2 | Forward(5’-3’) | TGGGCTATACTGGGAGCTACTG |
|  | Reverse(5’-3’) | GGATGGAAAATGGATAAGGATGAT |
| NOTCH3 | Forward(5’-3’) | GTGATCGGCTCGGTAGTAATG |
|  | Reverse(5’-3’) | ACAACGCTCCCAGGTAGTCA |
| NOTCH4 | Forward(5’-3’) | GTCGCTCACCAACGTAACCA |
|  | Reverse(5’-3’) | ACTCCGTATCTTCCTCGCATT |
| JAG1 | Forward(5’-3’) | TCACGGGAAGTGCAAGAGTC |
|  | Reverse(5’-3’) | GTTTCACAGTAGGCCCCCTC |
| HEY1 | Forward(5’-3’) | ATACGGCAGGAGGGAAAGG |
|  | Reverse(5’-3’) | GCGATGTGCGGGTGATG |
| DNMT1 | Forward(5’-3’) | GAGGAGGGCTACCTGGCTAA |
|  | Reverse(5’-3’) | CTCCATCGGACTTGCTCCTC |
| DNMT3A | Forward(5’-3’) | CTTTTGCGTGGAGTGTGTGG |
|  | Reverse(5’-3’) | CGGATGGGCTTCCTCTTCTC |
| DNMT3B | Forward(5’-3’) | AAGTCGAAGGTGCGTCGTG |
|  | Reverse(5’-3’) | TTCCAGGCTGCTCTTGTTGT |
| DNMT3L | Forward(5’-3’) | AGCATGGACGTGATTTTGGT |
|  | Reverse(5’-3’) | AGCATGGACGTGATTTTGGT |

**Supplementary Table 2. Sequences of shRNA Against Specific Target**

| sh-SIRT6 | 5’-3’ | CACCCGGATCAACGGCTCTAT |
| --- | --- | --- |
| sh-NOTCH1 | 5’-3’ | GCCGAACCAATACAACCCTCT |
| sh-NOTCH2 | 5’-3’ | GCAAGAATTGTCAGACAGTAT |
| Sh-DNMT1 | 5’-3’ | CGAGAAGAATATCGAACTCTT |
| sh-NC | 5’-3’ | TTCTCCGAACGTGTCACGT |
